# Clinical study on acupuncture treatment of hypertension with hyperactivity of liver yang

**DOI:** 10.1097/MD.0000000000025668

**Published:** 2021-04-30

**Authors:** Jiaojuan Wu, Xudong Zhang, Jiping Zhao, Yanjun Xue, Pengcheng Yu, Xiaoli Wu, Qingguo Liu

**Affiliations:** a School of Acupuncture-Moxibustion and Tuina, Beijing University of Chinese Medicine; b Dongzhimen Hospital, Beijing University of Chinese Medicine, Beijing, China.

**Keywords:** acupoint diagnosis, acupuncture, hypertension, randomized controlled trial

## Abstract

**Introduction:**

Hypertension can lead to different degrees complications of cardiovascular and cerebrovascular, and increase the risk of sudden death. Acupuncture has become a complementary alternative therapy for hypertension because of its antihypertensive and nontoxic side effects. However, there is still lack of evidence-based medicine evidence for an effective acupuncture antihypertensive prescription. The purpose of this study is to evaluate the effect of a special acupuncture prescription on hypertension with hyperactivity of liver yang.

**Methods:**

In this randomized controlled trial, we will recruit 56 hypertensive patients with hyperactivity of liver yang. Then the patients will be randomly divided into control group and experimental group. The control group will be treated with western medicine, and the experimental group will be treated with medicine combined with acupuncture. The intervention will last 4 weeks. The indices will be collected before acupuncture, after acupuncture, and 2 weeks after acupuncture. The primary outcome will be 24-hour ambulatory blood pressure. The secondary outcomes will be clinic blood pressure, anxiety and depression score, and the syndrome score of hyperactivity of liver yang. The auxiliary indicators will be blood pressure load values and salt sensitivity risk rate. The exploratory indicator will be acupoint diagnosis. The safety evaluation indicator will be incidence of adverse events.

**Discussion:**

The results of this study will provide favorable evidence for the efficacy and safety of acupuncture in reducing blood pressure, and explore the positive reaction acupoints which related to hypertension.

## 1 Introduction

Hypertension is one of the chronic diseases that seriously endanger human health. A study on hypertension epidemiology in 90 countries shows that the awareness rate of hypertension in adults is only 46.5%, the treatment rate is 6.9%, and the proportion of good blood pressure control is only 13.5%.^[1]^ The prevention and treatment of hypertension is still haunting the world. Most of the time, the treatment of hypertension is lifelong medication. The long-term use of antihypertensive drugs will bring damage of gastrointestinal, liver, and kidney function. In addition, antihypertensive drugs have the disadvantages of poor compliance and high cost of lifelong medication. Therefore, in recent years, in many countries’ hypertension treatment guidelines, it is mentioned to strengthen the intervention of non-drug therapy. In the review and analysis of clinical research report on acupuncture published by the World Health Organization,^[2]^ essential hypertension is definitely recommended as a disease for acupuncture treatment. This is obviously a confirmation of the antihypertensive effect of acupuncture. Acupuncture, as a characteristic therapy of traditional Chinese medicine (TCM), has the advantages of green and safety, and can adjust the body in multiple levels. With the international development of acupuncture, it has great potential and space in the prevention and treatment of global hypertension.

The synergism and attenuation effects of acupuncture on hypertension is a research hotspot at present. Whether it can produce better therapeutic effect on hypertension is the focus of our research. Some literatures have compared the clinical trials of different acupuncture prescriptions in the treatment of hypertension, and found that the antihypertensive effect of acupuncture combined with medicine is better than that of Western medicine alone. And the acupuncture prescription following the principle of dialectical treatment has obvious antihypertensive effect, otherwise, the effect are not good.^[3]^ Therefore, it is necessary to find a suitable prescription for acupuncture to reduce blood pressure, and strengthen the synergism and attenuation effects of acupuncture by combining with western medicine.

The purpose of this study is to evaluate the efficacy and safety of a special acupuncture prescription on hypertension with hyperactivity of liver yang (HLY), and to explore the positive reaction acupoints of patients with hypertension.

## 2 Method

### 2.1 Experimental design

**Figure 1:**
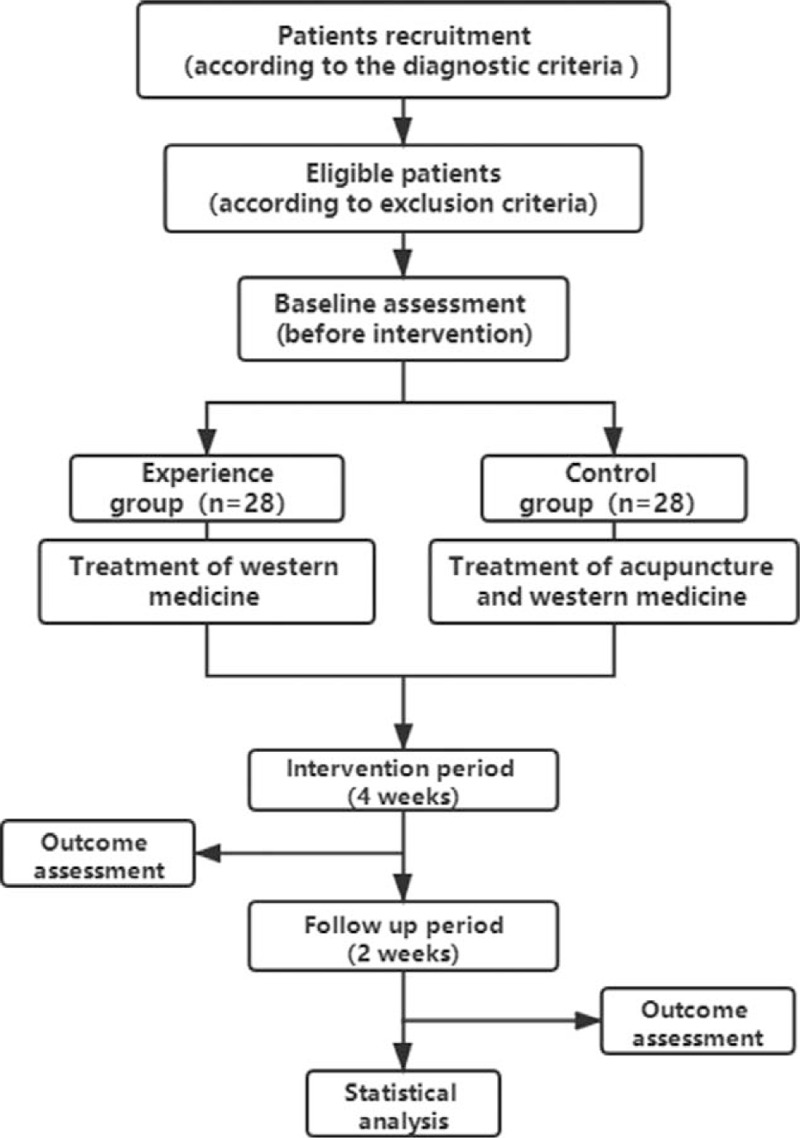
Flow chart of the study design.

This is a single-center, single-blind, randomized, controlled, parallel group clinical trial. The selected patients will be divided into control group and experimental group according to the ratio of 1:1. The latter will receive acupuncture treatment for 4 weeks on the basis of the former. The data will be collected before acupuncture, after acupuncture, and 2 weeks after acupuncture. The process is shown in Figure 1.

## 3 Participants

### 3.1 Source of participants

The participants in this study will be recruited from Dongzhimen Hospital of Beijing University of Chinese Medicine and its surrounding communities.

### 3.2 Diagnostic criteria

#### 3.2.1 Diagnostic criteria of western medicine

The diagnosis criteria of essential hypertension will be in accordance with the 2018 revision of the guidelines for prevention and treatment of hypertension of China, revised by the China hypertension Revision Commission.^[4]^ Without antihypertensive drugs, hypertension will diagnosed when systolic blood pressure (SBP) of clinic blood pressure (CBP) ≥140 mmHg and/or diastolic blood pressure (DBP) of CBP ≥90 mmHg are measured 3 times (not on the same day). The patients who had the history of hypertension and were taking antihypertensive drugs will also be diagnosed with hypertension. The diagnostic criteria of ambulatory blood pressure monitoring (ABPM) for hypertension are: 24 hour average SBP/DBP ≥130/80 mmHg; daytime SBP/DBP ≥135/85 mmHg; nighttime SBP/DBP ≥120/70 mmHg. The diagnostic criteria of home blood pressure monitoring (HBPM) ≥135/85 mmHg, which corresponded to 140/90 mmHg in CBP.

According to whether participants has cardiovascular risk, including target organ damage, cardiovascular risk factors, diabetes, and clinical complications, they will divided into 4 levels: low risk (without risk factors), medium risk (with 1–2 risk factors), high risk (with ≥3 risk factors), very high risk (with target organ damage, chronic kidney disease stage 3, or diabetes).

#### 3.2.2 TCM diagnostic criteria

According to the expert consensus on diagnosis and treatment of hypertension in TCM (2019 edition),^[5]^ the guiding principles for clinical research of new Chinese medicine in the treatment of hypertension^[6]^ and the criteria for diagnosis and treatment of TCM syndromes (2017 edition),^[7]^ the TCM diagnostic criteria for HLY will be formulated. On the basis of the main symptoms, those who have ≥3 secondary symptoms at the same time combined with tongue and pulse conditions, can be diagnosed as HLY.

To guarantee the consensus on inquiry method, judgment of syndrome type and record method, the clinical data will be collected by unified questionnaire and unified criteria. It will be reviewed by the deputy chief physician and above. If the judgment of syndrome type is inconsistent, 2 chief physicians of TCM will assist in the diagnosis.

### 3.3 Inclusion criteria

1. Age: 18 to 65 years, both male and female;
2. Met the Western diagnostic criteria of essential hypertension. For patients without antihypertensive drugs, SBP: 140 to 179 mmHg and/or DBP: 90 to 109 mmHg. For patients with antihypertensive drugs, hypertension should be stabilized at a normal high value. The patients who took medicine used ≤2 antihypertensive drugs for >2 months.
3. Confirmed TCM diagnosis of HLY in hypertension.
4. Not receives acupuncture or Chinese herbal medicine treatment in recent 1 month.
5. Good compliance with the observation and evaluation of researchers.
6. Informed consent is signed by the patient himself or his immediate family members.

### 3.4 Exclusion criteria

1. Secondary hypertension, such as aldosteronism, pheochromocytoma, Cushing syndrome, gestational hypertension, obstructive sleep apnea hypopnea syndrome, and so on.
2. Accompanied by other serious cardiovascular and cerebrovascular diseases, nephropathy, retinopathy, peripheral vascular diseases, diabetes, blood diseases, and so on.
3. There were skin diseases and severe skin damage near the acupoints.
4. Mental disorders, pregnant, and lactating women.
5. Participating in other clinical trials.

### 3.5 Elimination of participants and their treatment

#### 3.5.1 Elimination criteria

1. The patients who do not complete the treatment according to the regulations or the data are incomplete, which affect the later evaluation.
2. Noncompliant patients, those who withdraw from the research by themselves.
3. Unauthorized use of drugs and treatment methods other than those specified in this protocol.
4. During the trial, patients with serious adverse reactions or complications. The patients are intolerant of acupuncture therapy, or should not continue to receive treatment.
5. Patients lost to follow-up.

#### 3.5.2 Treatment of elimination participants

1. When the patients falls off, we will contact by telephone, e-mail, and so on, as far as possible to ask for the reason. Record the time of the last treatment and complete the assessment items that can be completed.
2. Patients who withdraw from the trial due to adverse reactions or ineffective treatment will take corresponding measures according to their actual situation.
3. Intention analysis will be conducted after the end of the trial, then the reason and time of withdrawal will be recorded in detail. Those who had more than half course of treatment will be included in the statistical analysis of curative effect.

### 3.6 Termination criteria

1. Patients with serious adverse reactions, special physiological changes, and other unexpected events during the study period.
2. Patients with severe complications, disease progression, and other critical illness during the study period.
3. Patients who did not obey the treatment.

## 4 Research methods

### 4.1 Random scheme and random hiding

This study will adopt a single-center, randomized, single blind, simple positive drug control method. Simple random number method will be used for grouping. Input the expected sample size into the statistical software package PEMS3.1, and get the sequence number. Then make a random card and add an opaque envelope to seal it. When the qualified patients enter the trial, the envelopes will be opened according to the order of entry, and they will be grouped according to the information on the random card.

### 4.2 Estimation of sample size

The purpose of this study is to observe that the antihypertensive effect of acupuncture combined with medicine is not inferior to that of Western medicine alone. Therefore, the sample calculation is based on the sample content calculation formula of noninferiority test.^[8]^ The calculation result is 22.57, that is, the experimental group and the control group need about 23 patients, respectively. According to the shedding rate of 20%, a total of 56 patients will be recruited.

### 4.3 Implementation of blind method

Due to the particularity of acupuncture clinical operation, this study will adopt a single-blind method, so only the efficacy evaluators and data statisticians will be blinded.

### 4.4 Ethical review

Before recruiting patients, this study had passed the ethical approval of the medical ethics committee of Dongzhimen Hospital of Beijing University of Chinese medicine (approval number: DZMEC-KY-2020-06). The registration was completed in China Clinical Trial Registration Center (Registration Number: ChiCTR2000037444). All patients will be fully informed about the trail and given enough time to decide whether to participate in the study. All patients will be asked to sign an informed consent form if they agree to participate in the study.

## 5 Treatment plan

### 5.1 Acupuncture group

This group of patients will receive Western medicine treatment (each patient's specific medication should follow the advice of cardiovascular doctors), and will also receive acupuncture treatment at the same time. Record the type, dose, and frequency of drugs taken by each patient. The frequency of acupuncture will be once a day, 30 minutes each time, 5 times a week (2 days off), a total of 4 weeks.

#### 5.1.1 Acupoint selection

Acupoint selection follows the semi standardized characteristics of acupuncture clinical research. Same main acupoints and reinforcing reducing manipulations will be used in this group, and the matching acupoints will be selected according to the principle of individualization.

Main acupoints: Taichong (LR 3-double); Baihui (DU 20); Taixi (KI 3-double); sanyinjiao (SP 6-double); Zusanli (ST 36-double).

Matching acupoints: headache: Fengchi (GB 20-double), Shuaigu (GB 20-double), Touwei (ST 8-double); insomnia: Sishencong (EX-HN), Benshen (GB 14-double), Shenting (DU 24), Shenmen (HT 7-double); excessive phlegm dampness syndrome: Yinlingquan (SP 9-double), Fenglong (ST 40-double); insufficiency of primordial QI: Qihai (RN 6), Guanyuan (RN 4).

#### 5.1.2 Operation

It will be positioned according to the standard position of WHO in the Western Pacific Region.^[9]^ Acupuncture operation will be carried out according to the operation method of Acupuncture, a textbook of the 13th five-year plan of China.^[10]^ The manipulations will be carried out according to the operation standard of twirling reinforcing reducing manipulation which agreed by our research group. Andy brand acupuncture needle (0.30 × 40 mm × 100 pieces/box) will be used for acupuncture. Patients in supine or prone position, doctors choose acupoints according to the patient's personal situation. First of all, after local disinfection, then acupuncture to the appropriate depth. Make the acupoints De Qi (the patient's description are judged as “De Qi” by “acid, numbness, heaviness and distension”). Then, with the right hand as the needling hand, 1 minute reinforcing manipulation at Taichong (LR 3-double) and 1 minute reducing manipulation at Sanyinjiao (SP6-double) will be performed. Finally, the needles will be kept for 30 minutes.

Baihui (DU 20), Taixi (KI 3-double), Zusanli (ST 36-double), and other matching acupoints will only retain needles with no manipulations. Twirling reinforcing reducing manipulations are as follows. Twirling reducing manipulation: the thumb exerts the heavy force to twist backward, and the light force to forward return, twirling range is 270 to 360 °/time, 60 times/minute, continuous rotation for 1 minute, retention for 29 minutes, a total of 30 minutes. Twirling reinforcing manipulation: the thumb exerts the heavy force to twist forward, and the light force to backward return. The twisting range is 270 to 360 °/time, 60 times/minute, continuous rotation for 1 minute, retention for 29 minutes, a total of 30 minutes.

The acupuncture operators involved in this study are postgraduates majoring in acupuncture and moxibustion, and they have obtained the qualification certificate of licensed doctor for >2 years.

### 5.2 Treatment plan of control group

The patients in the control group will be treated with antihypertensive drugs of western medicine, without changing the original western medicine treatment (the specific medication of each patient should follow the advice of cardiovascular specialist). And the patients will be instructed to take medicine regularly.

## 6 Efficacy index

### 6.1 Main outcome

Ambulatory blood pressure monitoring: 24 hours ambulatory blood pressure is the “criterion standard” to evaluate the antihypertensive effect.^[11]^ In this study, the 24 hours ambulatory blood pressure monitor (DMS-ABP) made by American Tim will be used for data collection. Ambulatory blood pressure will be measured in patients’ nondominant arm to reduce the impact of activity on blood pressure measurement. The detection time of each patient should be >23 hours. At the same time, the effective reading is >70%. There are at least 20 daytime blood pressure readings and 6 nighttime blood pressure readings.

Ambulatory blood pressure monitoring will be evaluated in the first week (baseline period), the fifth week (after acupuncture period), and the seventh week (after follow-up period).

### 6.2 Secondary outcomes

1. Clinic blood pressure: This index is used to assist the observation of blood pressure change trend of patients. The same doctor will use the same blood pressure meter in the same period on the same day. The blood pressure will be measured with Omron brand blood pressure meter (HEM-8711). After 5 minutes’ rest in a quiet environment, the blood pressure of the patient's right upper arm will be measured. Measure twice and take the average value. If the difference between the 2 times is >5 mmHg, the average value of 3 times shall be taken.
2. Hospital Anxiety and Depression Scale (HAD): HAD is composed of anxiety scale and depression scale. The highest score of HAD is 42. The higher the score is, the worse the mental state of the patients is. The total score of 0 to 7 is normal, 8 to 10 is mild, 11 to 14 is moderate, and 15 to 21 is serious anxiety/depression. The patients’ mental and emotional health will be evaluated by the HADs.
3. Syndrome integral of HLY: This index is used to observe the changes of syndrome of patients. Referring to the symptom grading scale, the symptom grading scale of HLY will be formulated. The symptom grading scale is from the diagnostic and curative effect standard of traditional Chinese medicine (2017 Edition).^[7]^ The evaluation symptoms included dizziness, headache, irritability, blush, hot eyes, thirst, bitter taste, constipation, reddish urine, dysphoria in chestpalms-soles, tinnitus, insomnia, and lumbar debility. The highest score is 42, and the lowest score is 0. The higher the score, the more serious the symptom.

The secondary outcomes mentioned above will be evaluated in the first week (baseline period), the fifth week (after acupuncture period), and the seventh week (after follow-up period).

### 6.3 Auxiliary outcomes

1. Blood pressure load: blood pressure load is the percentage of times that SBP and DBP readings exceed the normal range respectively. The normal standard is 24- hour ambulatory blood pressure <130/80 mmHg, daytime blood pressure <135/85 mmHg, and nighttime blood pressure <120/70 mmHg. It is generally considered that normal load should be <5%.
2. Risk rate of salt sensitivity: In 2011, Italian scholars proposed that the risk rate of salt sensitivity can be preliminarily determined by the nighttime blood pressure drop rate (monitored by 24-hour ambulatory blood pressure) combined with 24-hour average heart rate.^[12]^ The specific assessment method: the blood pressure of the observed patients does not drop at night, that is, the patients with nondipper blood pressure. If their 24-hour heart rate is ≥70 beats/min, about 70% of them are salt sensitive in the saline infusion test, and define this type of patients as having high risk of salt sensitivity. Dipper blood pressure and 24-hour heart rate <70 beats/min are defined as low risk of salt sensitivity, whereas others are defined as medium risk of salt sensitivity.

The auxiliary outcomes mentioned above will be evaluated at the first week (baseline period) and the fifth week (after acupuncture period).

### 6.4 Exploratory indicators

Acupoint diagnosis: all patients will be given acupoint diagnosis. Diagnostic acupoints: acupoints with 12 meridians below elbow and knee joint (1 cm around acupoints). In addition, there are some back shu points, including Ganshu (BL 18), Danshu (BL 19), Xinshu (BL 15), Pishu (BL 20), Weishu (BL 21), and Shenshu (BL 23) (1 cm around the points).

Specific diagnosis methods (traditional manual detection): slide the thumb pulp within 1 cm of the acupoint, ① Feel the abnormal reaction of the skin and the superficial part of the acupoint with light strength; ② Detect the abnormal performance of the muscle with medium strength, including streak, tubercle, bulge, and hollow; ③ Detect the abnormal reaction of the deeper layer with slightly heavy strength; ④ Apply the force evenly, and review repeatedly, then record the results. The acupoints with positive reaction and their specific conditions will be recorded. Then the pain degree will be evaluated by the international commonly used visual analogue scale (VAS), and the VAS score will be recorded. The acupoint diagnosis will conducted be in the first week (baseline period) and the fifth week (after acupuncture period) of the eligible participants.

### 6.5 Safety indicators

#### 6.5.1 Safety assessment

The experiment will observe the adverse events of the participants during the experiment (including acupuncture related events such as bleeding, hematoma, pain of acupuncture site, fainting during acupuncture treatment, high blood pressure, among others, and nonacupuncture-related events such as cold, cough, aggravation of pre-existing diseases, and so on). And fill in the adverse event report form (record time, location, symptom, degree, duration, treatment method, treatment result, among others, and sign the name and date). At the end of the treatment, the causes of each event will be analyzed and summarized carefully.

#### 6.5.2 Treatment of adverse events

If there are adverse reactions in the process of acupuncture, it is necessary to deal with them timely and properly until the patient's condition is stable. Those with abnormal laboratory tests will be tracked until they return to normal. Researchers will need to analyze whether the adverse events are related to the trial interventions, and decide whether to discontinue the trial based on the severity of the adverse events. For those with serious adverse reactions, relevant departments will be consulted immediately. And we will report to the ethics committee in the first time (within 2 hours).

## 7 Quality control and assurance

This study will control bias from the following aspects to improve the quality of research.

1. The participants will come from hospitals and surrounding communities to avoid hospital selection bias.
2. Acupuncture treatment, blood pressure measurement, and acupoint diagnosis will be operated by 3 doctors respectively (each item will operated by the same doctor), so as to avoid bias caused by doctors with different qualifications.
3. To reduce the measurement bias, the blood pressure measuring instrument recommended in Chinese guidelines for the prevention and treatment of hypertension (revised version 2018) will be used.
4. The data analysis team will be entrusted to analyze the data, and the research group will publish the real and complete results to eliminate the publication bias.

## 8 Data management

All information related with this trial will be recorded in CRFs by a trained and qualified investigator. No correction of completed CRFs is allowed once completed. The record of CRFs will be reviewed by the clinical inspector. To ensure the accuracy of the data, 2 investigators will input and proofread the data respectively. In addition, all papery documents and electronic versions of the CRF will be preserved in the secure research archives for 5 years after trial completing at the Dongzhimen Hospital Affiliated to Beijing University of Chinese Medicine and only can be viewed by the research team.

## 9 Statistical analysis

SPSS 20.0 statistical software will be used for data statistics (SPSS Inc, Chicago, IL). The frequency percentage will be used to describe the demographic data. For the measurement data with normal distribution and homogeneous variance, mean ± standard deviation (\bar{x} (Latex code) ± s) will used to describe the data. Paired and independent sample *t* test will used for intragroup and intergroup comparison respectively. For the measurement data of non-normal distribution, nonparametric test will be used. The blood pressure value will be analyzed by repeated measurement analysis of variance. In the correlation analysis of 2 theoretically related continuous variables, Pearson correlation coefficient model will be used if the data meet the normal distribution, and Spearman correlation coefficient model will be used if the data are nonnormal distribution. *χ*^2^ test will be used for counting data. All tests will be bilateral; *P* < .05 means the difference is statistically significant. In the correlation analysis, 0 < R≤0.2 means that there is no correlation between variables; 0.2<R≤0.4 means that there is a low correlation between variables; 0.4<R≤0.8 means that there is a moderate correlation between variables; 0.8 < R≤1 means that there is a high correlation between variables.

## 10 Discussion

The hypertension patients included in this study are HLY, which accounted for a high proportion (24.1%) of hypertension patients.^[13]^ Therefore, it has important research significance. TCM believes that HLY belongs to a syndrome type of water failing to nourish wood, and deficiency in origin and excess in superficiality. Its pathogenesis is related to a series of pathological manifestations caused by the reverse ascending of Chong Channel in TCM. Therefore, Taichong (LR 3), Baihui (DU 20), Taixi (KI 3), Sanyinjiao (SP 6), and Zusanli (ST 36) are used as the main acupoints for adjustment. The purpose of these acupoints is to stabilize the reverse ascending of Chong Qi and guide the Qi downward. At the same time, these acupoints can also nourish yin to suppress yang, and regulate qi and blood. Finally, the goal of reducing blood pressure will be achieved. In addition, according to other symptoms and syndrome types of patients, matching acupoints will be selected to achieve better antihypertensive effect.

Our research group has been engaged in the research of twirling reinforcing reducing manipulation for a long time.^[14–15]^ Therefore, according to the theory of reinforcing deficiency and reducing excess, reinforcing and reducing manipulations on the main acupoints can achieve better therapeutic effect. The operation time of manipulation is 1 minute, and the time setting is in line with the clinical operation. The indicators of the research will be evaluated at most 3 times, and the scale is not large, so it will be collected smoothly. In addition, salt sensitivity of hypertension is common in China. Because it is difficult to popularize invasive saline infusion test on a large scale, clinical evaluation is difficult. Although the effectiveness of the salt-sensitive risk assessment method used in this study needs to be verified, it has good feasibility and promotion value. Therefore, it is worthy of further study.

This experiment focused on 1 type of hypertension. The highlight of the experimental design is to fix the main acupoints according to the characteristics of HLY, and select the matching acupoints according to patient's conditions. Therefore, it will be more effective in reducing blood pressure and is in line with clinical operation.

In addition, this design not only verifies the hypotensive effect of a special acupuncture prescription, but also explores the related theories of acupoints and viscera. This is also the unique feature of this clinical study. In *Lingshu · Hailun*, it is said that “Twelve meridians belong to the viscera internally, and connect to the extremities and joints externally.” Therefore, the physiological and pathological conditions of the viscera will be reflected in the relevant acupoints. Many animal experiments have confirmed this.^[16]^ Because acupoint diagnosis takes more time, there are few studies in the current clinical trials. Acupoint diagnosis is to verify the correlation between acupoints and viscera. This result will also provide reference for the adjustment of acupoint prescription of hypertension.

Of course, this study also has some limitations. For example, the study cannot achieve double-blind, which may have some impacts on the results; the follow-up time of patients may be short, and so on.

In summary, this study will be able to evaluate the effect of a special acupuncture prescription on hypertension with hyperactivity of liver Yang, and will provide experimental basis for the selection of acupoints for hypertension.

## Author contributions

Xudong Zhang and Jiaojuan Wu are the co-first authors. They contributed equally to this work. Jiping Zhao designed this study. Qingguo Liu guides the experiment. Xudong Zhang will perform acupuncture treatment. Jiaojuan Wu is responsible for acupoint diagnosis. Yanjun Xue is responsible for blood pressure measurement. Xiaoli Wu and Pengcheng Yu are responsible for recruitment. All authors have read and approved the final manuscript.

**Data curation:** Yanjun Xue, Pengcheng Yu, Xiaoli Wu.

**Methodology:** Jiaojuan Wu, Xudong Zhang.

**Project administration:** Jiping Zhao, Qingguo Liu.
